# Proposal for new diagnostic criteria for DIC from the Japanese Society on Thrombosis and Hemostasis

**DOI:** 10.1186/s12959-016-0117-x

**Published:** 2016-09-28

**Authors:** Hidesaku Asakura, Hoyu Takahashi, Toshimasa Uchiyama, Yutaka Eguchi, Kohji Okamoto, Kazuo Kawasugi, Seiji Madoiwa, Hideo Wada, DIC subcommittee of the Japanese Society on Thrombosis and Hemostasis

**Affiliations:** 1 Department of Internal Medicine (III), Kanazawa University School of Medicine, 13-1, Takaramachi, Kanazawa, 920-8641 Japan; 2 Department of Internal Medicine, Niigata Prefectural Kamo Hospital, 1-9-1 Aomicho, Kamo, Niigata 959-1397 Japan; 3 Department of Laboratory Medicine, National Hospital Organization Takasaki General Medical Center, 36 Takamatsu-Cho, Takasaki, Gunma 370-0829 Japan; 4 Department of Critical and Intensive Care Medicine, Shiga University of Medical Science, Seta Tsukinowa-cho, Otsu, Shiga 520-2192 Japan; 5 Gastroenterology and Hepatology Center, Kitakyushu City Yahata Hospital, 4-18-1, Nishihon-machi, Yahatahigashi-ku, Kitakyushu, Fukuoka 805-8534 Japan; 6 Department of Hematology, Teikyo University School of Medicine, 2-11-1 Kaga Itabashi-Ku, Tokyo, 173-8605 Japan; 7 Department of Clinical and Laboratory Medicine, Tokyo Saiseikai Central Hospital, 1-4-17, Mita, Minato-ku, Tokyo, 108-0073 Japan; 8 Department of Molecular and Laboratory Medicine, Mie University Graduate School of Medicine, Tsu, Mie 514-8507 Japan

**Keywords:** Disseminated intravascular coagulation, DIC, Diagnostic criteria, Suppressed-fibrinolytic-type DIC, Enhanced-fibrinolytic-type DIC

## Abstract

Disseminated intravascular coagulation (DIC) is a serious disease that, in the presence of underlying disease, causes persistent, generalized, marked coagulation activation. Early treatment based on an appropriate diagnosis is very important for improving patients’ prognosis, to which end diagnostic criteria play a key role. Several criteria have been proposed, but each has its strengths and weaknesses, and improved criteria are needed. Widespread use of coagulofibrinolytic markers has elucidated that the pathology of DIC differs greatly as a function of the underlying disease. Thus, discriminating use of DIC diagnostic criteria that take underlying diseases into account is important.

DIC diagnostic criteria that are well known in Japan include the Japanese Ministry of Health and Welfare’s old DIC diagnostic criteria (JMHW criteria), the International Society on Thrombosis and Haemostasis’s DIC diagnostic criteria (ISTH criteria), and the Japanese Association for Acute Medicine’s acute-stage DIC diagnostic criteria (JAAM criteria). Those criteria have their respective drawbacks: the sensitivity of the ISTH criteria is poor, the JAAM criteria cannot be applied to all underlying diseases, and the JMHW criteria have poor sensitivity in the case of infections, do not use molecular markers, and result in misdiagnosis. The Japanese Society on Thrombosis and Hemostasis’s newly proposed provisional draft DIC diagnostic criteria (new criteria) use diagnostic criteria classifications of “hematopoietic disorder type”, “infectious type”, and “basic type” based on the underlying pathology. For the hematopoietic disorder type the platelet count is omitted from the score, while for the infectious type, fibrinogen is omitted from the score. Also, points are added if the platelet count decreases with time. In the new criteria, molecular markers and antithrombin activity have been newly included, and as a countermeasure for misdiagnosis, 3 points are deducted if there is liver failure. In this paper, we discuss various problems encountered with DIC diagnosis, and we describe the new criteria together with the events that led to their creation.

These new diagnostic criteria take into account the underlying diseases of wide area, and we expect that they will serve clinicians well due to the above adaptations and improvements.

## Background

Disseminated intravascular coagulation (DIC) is a serious disease that, in the presence of underlying disease, causes persistent, generalized, marked coagulation activation and frequent formation of microthrombi in microvessels. Both coagulation activation and fibrinolytic activation are seen, but the severity of the fibrinolytic activation differs considerably as a function of the underlying disease(s). Progression of DIC causes decreases in hemostatic factors such as platelets and clotting factors and leads to consumption coagulopathy [1–5]. The two major symptoms of DIC are bleeding symptoms and organ symptoms, and the prognosis becomes very poor if the clinical symptoms become apparent. For that reason, it is ideal to initiate treatment of DIC before clinical symptoms manifest.

The Scientific Standardization Committee (SSC) of the International Society on Thrombosis and Haemostasis (ISTH) defined DIC as follows: “DIC is an acquired syndrome characterized by the intravascular activation of coagulation with loss of localization arising from different causes. It can originate from and cause damage to the microvasculature, which if sufficiently severe, can produce organ dysfunction” [6]. The view of the ISTH can be considered to represent the world’s general perception regarding DIC. In fact, the pathology of DIC complicated by sepsis or some other severe infection is accurately presented. However, serious bleeding symptoms may occur due to marked fibrinolytic activation, as in the case of DIC secondary to acute leukemia (especially acute promyelocytic leukemia), aortic aneurysm, giant hemangioma, placental abruption, and metastatic prostate cancer. Although organ symptoms are usually not seen, there is a problem in that consideration has not been given to DIC with severe bleeding symptoms [7, 8].

DIC is a serious condition, and early treatment based on an appropriate diagnosis is very important for improving patients’ prognosis, in which effective diagnostic criteria play a key role. In this paper, we discuss the diversity in DIC, as well as various problems encountered with DIC diagnosis, and describe the provisional draft DIC diagnostic criteria that have been proposed by the Japanese Society on Thrombosis and Hemostasis (JSTH) (the new criteria).

## Review

### DIC disease type classification

There are various DIC disease types, depending on the underlying disease. The concept of DIC disease type classification is important to understanding that diversity [9]. Marked coagulation activation is the primary pathology of DIC and seen in all cases, but in regard to other aspects, the pathology (especially the degree of fibrinolytic activation) differs considerably as a function of the underlying disease. The degree of fibrinolytic activation is controlled by plasminogen activator inhibitor-1 (PAI-1), which is one of the important factors characterizing DIC.

In suppressed-fibrinolytic-type DIC, coagulation activation is high, whereas fibrinolytic activation remains mild. This type of DIC is seen in cases complicated by sepsis. Lipopolysaccharide (LPS) and inflammatory cytokines act on the vascular endothelium, thereby enhancing production of fibrinolysis inhibitory factor PAI-1 and creating a potent state of inhibition of fibrinolysis. Dissolution of multiple microthrombi becomes difficult, increasing the risk of organ failure due to failure of the microcirculation, but bleeding symptoms are relatively mild. Laboratory findings include elevated levels of thrombin-antithrombin complex (TAT), soluble fibrin (SF), and prothrombin fragment 1 + 2 (F_1+2_), which are coagulation activation markers, but plasmin-α_2_-plasmin inhibitor complex (PIC), a fibrinolytic activation marker, is only slightly elevated [10, 11]. Other characteristic findings are relatively mild increases in fibrin/fibrinogen degradation products (FDP) and D-dimer, which reflect lysis of microthrombi. Intrinsically, α_2_-plasmin inhibitor (α_2_PI) is consumptively decreased in DIC. However, in suppressed-fibrinolytic-type DIC, α_2_PI is generally slightly below or almost normal because plasmin production is low, and α_2_PI is a protein that increases in inflammation. FDP and D-dimer are recognized as the most important markers for DIC diagnosis. However, in suppressed-fibrinolytic-type DIC, it is not unusual for their elevation to be mild, leading to concern that diagnosis of DIC might be delayed if too much emphasis is placed only on these markers. Conversely, early diagnosis is possible if attention is paid to elevation of TAT and SF as blood markers, together with a decrease in the platelet count with time. Because of the inflammatory reaction, fibrinogen often does not decrease.

Enhanced-fibrinolytic-type DIC is characterized by marked fibrinolytic activation that is out of balance with coagulation activation. Characteristic patients have underlying diseases such as acute promyelocytic leukemia (APL), aortic aneurysm, and prostate cancer. PAI-1 is hardly elevated, while the strong fibrinolytic activation and hemostatic plugs (thrombi for hemostasis) are readily dissolved. As a result, bleeding symptoms are likely to be severe, but organ failure is almost never seen. In APL and some cancers, annexin II is involved in strong fibrinolytic activation [12, 13]. Laboratory findings include marked increases in the coagulation activation markers, TAT (SF, F_1+2_), and the fibrinolytic activation marker, PIC, while FDP and D-dimer (especially FDP) are also increased [10, 11]. It is also characteristic for fibrinogen and α_2_PI to be markedly decreased. Even if the platelet count is only slightly decreased, caution is needed in regard to potential massive bleeding. Furthermore, when treating enhanced- fibrinolytic-type DIC, it is not unusual for administration of only a heparin to promote bleeding, and in such cases it is effective to concomitantly administer nafamostat mesylate (a potent anti-thrombin agent that also has anti-plasmin activity) or tranexamic acid with a heparin [14–17].

Balanced-fibrinolytic-type DIC is characterized by balance between the coagulation and fibrinolytic activations and is thus an intermediate pathology between the two types of DIC described above. With the exception of advanced cases, bleeding symptoms and organ symptoms are relatively rare. Although this type is seen in many DIC cases with solid cancers, in the case of some cancers such as prostate cancer and blood vessel-associated malignant tumors, the disease type is enhanced-fibrinolytic-type DIC.

Gando et al. reported that DIC seen at the time of trauma is DIC with the fibrinolytic phenotype in which the initial fibrinolytic activation is high, but at 24–48 h post-trauma, the pathology changes to a thrombotic phenotype due to the activity of PAI-1 [18, 19]. For DIC caused by trauma, the fact that tranexamic acid is used only at the time of enhanced-fibrinolytic-type DIC can be considered to be the point. The concept is that DIC with the fibrinolytic phenotype is close to enhanced-fibrinolytic-type DIC, while DIC with the thrombotic phenotype is close to suppressed-fibrinolytic-type DIC.

The commonly used conventional animal models of DIC are induced with LPS or tissue factor (TF) (especially LPS), but the actual situation is that little conscious distinction has been made between them as the same DIC model. However, our group recently showed that the pathology differs greatly depending on the DIC inducer used [9]. The LPS-induced DIC model is characterized by a state of suppression of fibrinolysis due to markedly elevated PAI-1 activity and only mild elevation of D-dimer. It is easy to pathologically demonstrate multiple microthrombi. Whereas organ disorders such as hepatorenal disorder are advanced, bleeding symptoms are hardly seen, even though the platelet count and fibrinogen are markedly reduced [20]. Meanwhile, the TF-induced DIC model is characterized by only a mild increase in PAI-1 activity, while D-dimer is sharply increased, reflecting the fact that there is adequate fibrinolytic activation. It is difficult to pathologically demonstrate microthrombi due to enhanced thrombolysis. Interestingly, whereas there is almost no hepatorenal failure, severe hematuria is seen as a bleeding symptom. Moreover, the high degree of fibrinolytic activation results in progression of not only fibrin degradation, but also fibrinogen degradation [21]. In this way, it can be thought that the LPS-induced DIC model is similar in pathology to clinical suppressed-fibrinolytic-type DIC, while the TF-induced DIC model is pathologically similar to enhanced-fibrinolytic-type - balanced-fibrinolytic-type DIC.

### Representative DIC diagnostic criteria

Three DIC diagnostic criteria are well known in Japan: the Japanese Ministry of Health and Welfare’s DIC diagnostic criteria (JMHW criteria), the ISTH’s DIC diagnostic criteria (ISTH criteria), and the Japanese Association for Acute Medicine’s DIC diagnostic criteria (JAAM criteria) [6, 22, 23] (Table 1).

**Table 1:** Comparison of existing DIC diagnostic criteria

	JMHW	ISTH	JAAM
Underlying disease Clinical symptoms	1 p bleeding: 1 p organ failure: 1 p	0 p(essential) 0 p 0 p	0 p(essential) SIRS score ≥3: 1 p
Platelet count (X10^4^/μL)	8 < − ≤12 : 1 p 5 < − ≤8 : 2 p ≤5 : 3 p	5–10 : 1 p <5 : 2 p	8 - ≤12 or >30 % reduction/24 h: 1 p <8 or >50 % reduction/24 h: 3 p
Fibrin-related marker	FDP (μg/ml) 10 ≤ − <20: 1 p 20 ≤ − <40: 2 p ≥40 : 3 p	FDP, D-dimer, SF moderate increase: 2 p strong increase: 3 p	FDP (μg/ml) 10 ≤ − <25: 1 p ≥25 : 3 p
Fibrinogen (mg/dl)	100 < − ≤150: 1 p ≤100: 2 p	<100: 1 p	None
PT	PT ratio 1.25 ≤ − <1.67: 1 p ≥1.67: 2 p	Prolonged PT(sec) 3–6: 1 p >6: 2 p	PT ratio ≥1.2: 1 p
Diagnosis of DIC	≥7 p	≥5 p	≥4 p

p: points

JMHW: JMHW criteria; ISTH: JMHW criteria; JAAM: JMHW criteria; PT: prothrombin time

JMHW criteria: When there is leukemia/related diseases, aplastic anemia, or marked bone marrow megakaryocyte reduction, such as after administration of an anti-tumor agent, and a high degree of thrombocytopenia, the bleeding symptom and platelet count items should be calculated as 0 points, and DIC is diagnosed if the score is ≥4 points

Those criteria have their respective drawbacks, such as the poor sensitivity of the ISTH criteria and that the JAAM criteria cannot be applied to all underlying diseases (e.g., DIC complicated by a hematopoietic malignancy cannot be diagnosed). To date, the JMHW criteria have the longest history and a solid reputation.

However, the JMHW criteria have also been noted to have various problems because they have poor sensitivity in the case of infections, do not use molecular markers that reflect coagulation activation (which is at the heart of DIC), and result in misdiagnosis of liver failure. With the aim of developing better DIC diagnostic criteria, the SSC/DIC subcommittee of the Japanese Society on Thrombosis and Hemostasis (JSTH) held numerous discussions over the years, but a consensus was not reached. For that reason, in July 2012, the JSTH established a committee charged with creating DIC diagnostic criteria.

That committee also carried out numerous in-depth discussions, both in meetings and via e-mail exchanges. The committee had 13 members, consisting of 6 from the SSC/DIC subcommittee and 7 others. The members represented various medical fields: internal medicine, surgery, obstetrics and gynecology, pediatrics, clinical laboratory medicine, and emergency medicine. In addition, the SSC/DIC subcommittee members put in a collective effort and carried out a thorough literature search.

This culminated, in October 2014, with publication of the JSTH’s provisional draft DIC diagnostic criteria (hereinafter, new criteria) [24]. The criteria are considered to be provisional, because they are likely to be modified on the basis of the results of future studies. Since that paper was published in a Japanese journal, the new criteria remain largely unknown internationally. We hope to remedy that situation through publication of the present English manuscript.

### Basic concept of these DIC diagnostic criteria

Three main methods exist for creating diagnostic criteria. The first method is creation of criteria based on the definition of DIC. The ISTH’s overt DIC criteria are equivalent to this. Since DIC is a domain that is evidence-poor, the approach is to try to create criteria by defining the portions for which there is no evidence [6]. The second method aims to create criteria that reflect the prognosis. In fact, most existing diagnostic criteria reflect the prognosis. No DIC diagnostic criteria have been drawn up based on prognosis as the endpoint, but there are a number of papers of DIC diagnostic criteria that secondarily reflect the prognosis [25–28]. The third method is to compile cases of DIC diagnosed by experts and then create diagnostic criteria. The JMHW criteria used this approach [29, 30].

Because there are no specific markers for DIC, it makes sense to diagnose DIC using a scoring method that combines multiple markers that show characteristic changes in DIC. The DIC clinical practice guidelines of the United Kingdom, Japan, and Italy that have been published as academic papers in English all recommend using a scoring method to diagnose DIC [5, 31–33]. The JMHW criteria, ISTH criteria, and JAAM criteria all carry out diagnosis using a scoring method [6, 22, 23] (Table 1). Japan has a long history of DIC clinical trials of various drugs that have been performed using the JMHW criteria. For that reason, it can be thought that, even when the objective is to create new, improved diagnostic criteria, it would be inappropriate to create totally new criteria, and that the JMHW criteria should be kept as the core. Thus, even the new criteria must inevitably use a scoring method.

The background of creation of the JAAM criteria was that diagnosis by the JMHW criteria was often too late in the fields of emergency medicine and surgery [29]. However, it was often pointed out that, while the sensitivity of the JAAM criteria is high, their specificity is low [27], and they also do not incorporate the molecular markers associated with coagulation activation that reflect the nature of DIC. Verification of the JAAM criteria was performed by collecting cases in the emergency medicine field, and it was named the acute phase DIC diagnostic criteria, but originally the criteria were positioned only for the emergency medicine field (not all acute phase). The ISTH criteria were developed by modeling the Japanese JMHW criteria, but they were even less sensitive than the JMHW criteria [23].

Today, we know from analyses using molecular markers that the pathology of DIC differs greatly depending on the underlying disease [7, 9–11]. This means that there are limitations on the ability of any one set of criteria to diagnose all presentations of DIC. It can be thought that the DIC criteria should be selected and used in the light of a patient’s underlying disease(s). However, because the numerous classifications of underlying diseases might complicate DIC diagnostic criteria, diagnosis should be performed using a set of criteria for the basic type and then adding different diagnostic items that are appropriate for the hematopoietic disorder type and infectious type of DIC. In particular, since the JMHW criteria are excellent for blood diseases but have weak diagnostic capacity for infectious diseases, there is a need for modification to address this point.

With the JMHW criteria, it is not uncommon for a coagulation abnormality that is accompanied by liver disease to be misdiagnosed as DIC, and that has to be considered.

The two major clinical symptoms of DIC are bleeding symptoms and organ symptoms. However, the clinical symptoms are non-specific, and it can be difficult to determine if they are symptoms due to an underlying disease or a complication other than DIC or are symptoms caused by DIC. Also, if no symptoms manifest and DIC is thus not diagnosed, that is an obstacle to early diagnosis of the disease. It can thus be thought that the clinical symptoms used in the JMHW criteria should be removed from the diagnostic criteria.

### Significance of various markers for DIC diagnosis

#### FDP, D-dimer

FDP and D-dimer have great significance in the diagnosis of DIC, and they are, in fact, included as important test items in almost all DIC diagnostic criteria [6, 22, 29]. However, it must be kept in mind that, while FDP and D-dimer are high in sensitivity, they are low in specificity. For example, these markers are often elevated even in such diseases as deep vein thrombosis, pulmonary thromboembolism, massive hydrothorax/ascites, and large subcutaneous hematomas [34–36].

Since FDP and D-dimer do not necessarily coincide with the molecular species of interest, there is medical significance in measuring both. For example, not only fibrin but also fibrinogen is broken down in DIC [37], and fibrinogen degradation is increased when the fibrinolytic system is highly activated. FDP increases markedly, but D-dimer rises only moderately. This results in a dissociation phenomenon between FDP and D-dimer (i.e., the D-dimer/FDP ratio decreases) [13, 37–39]. However, we should refrain from aimlessly measuring both FDP and D-dime.

In the case that fibrin/fibrinogen degradation is advanced due to strong fibrinolytic activation, the reaction with the D-dimer fraction may be reduced depending on the D-dimer reagent that is used [39]. Accordingly, for the DIC diagnostic criteria, it can be thought that emphasis should be placed on FDP rather than D-dimer.

#### Platelet count

As noted earlier, the platelet count cannot be used in the diagnostic criteria for diagnosis of the hematopoietic disorder type, and sufficient caution is necessary in this regard.

Except for the hematopoietic disorder type, the platelet count, like FDP and D-dimer, is an important test item for DIC diagnosis. However, there are also many patients with a decreased platelet count that is not due to DIC, and it is necessary to raise awareness regarding diseases that need to be differentiated from DIC. A decreased platelet count has high sensitivity for diagnosis of DIC, but it can be said that its specificity is low [23, 27, 31, 40].

Changes in the platelet count with time are also important. For example, even if the platelet count is above 12 × 10^4^/μL, DIC may be present if the count decreases with time. For this reason, there is significance in assigning a score to the thrombocytopenia rate, separate from the platelet count [41, 42].

#### Fibrinogen

For diagnosis of DIC, fibrinogen is a marker with high specificity, but low sensitivity [29, 30, 43–45]. Especially in inflammatory diseases, fibrinogen does not decrease even in patients thought to have DIC, and in some cases it actually increases [46]. The JAAM criteria were initially created using fibrinogen for DIC diagnosis at ≥5 points, but in actual application, fibrinogen could not be shown to have diagnostic significance. The criteria were thus modified by eliminating fibrinogen for DIC diagnosis at ≥4 points [29]. This is probably because the validation of JAAM criteria was performed in many cases with concurrent infection. There are underlying diseases for which fibrinogen is valuable as a marker. For example, fibrinogen readily decreases in patients with hematopoietic malignancies, obstetric complications, head trauma, aortic aneurysm, and solid cancers, and is an important finding [47–51].

Against this background, one approach would be to change the test items in the diagnostic criteria to fit each underlying disease. That is, in the case that the infectious type is the underlying disease, it is desirable to exclude fibrinogen, which fluctuates as an acute-phase reactive protein, from the score.

#### Prothrombin time (PT)

PT can reflect organ failure, and the prognosis is poor in patients with an infection and a prolonged PT [29, 30, 52–55]. On the other hand, since PT is also prolonged in liver disease and vitamin K deficiency, it is not a characteristic marker for DIC.

Most studies that investigated the sensitivity and specificity of PT in the diagnosis of DIC examined cases of infection, and almost none examined other underlying diseases [29, 30, 43, 44, 46]. Ordinarily, verification of significance of PT in diagnostic criteria should be performed in various underlying diseases such as infectious disease, hematopoietic malignancy, and solid cancer. Because PT has been used for many years and has a proven track record, it was decided to include it in the new criteria.

There is a problem as to the notation that should be used for PT in the new criteria, i.e., PT ratio or INR. INR values using different PT reagents converge in patients on warfarin but not in patients with liver disease or DIC. INR (liver) has been proposed [56, 57], and these values converges in liver disease but not in DIC. In the case of using PT for DIC diagnosis, the PT ratio must be used at present. However, given the current situation that the INR notation is widely used, if the ISI of the PT reagent is close to 1, it can be thought that INR can be substituted for the PT ratio.

#### Molecular markers of coagulation activation

TAT, SF, and F_1+2_ are molecular markers that reflect coagulation activation, which can be said to be at the heart of DIC. Incorporation of these molecular markers in DIC diagnostic criteria can be expected to improve both the sensitivity and specificity of the criteria [58–63]. Furthermore, TAT and SF are significant in that, if both are completely normal, then they can be used for exclusion of the diagnosis, that is, to determine that DIC is not present. At present, many institutions still do not measure these markers in-hospital, but that can be expected to change if they are included in the DIC diagnostic criteria. Even institutions that do not perform in-hospital assays will be able to confirm the test results at a later date, and it can be thought that this will help reduce misdiagnoses.

On the other hand, we can anticipate that contrary opinions will also be expressed, such as that there are many institutions that would not get the results for these molecular markers on the same day, and especially in pediatric departments, which have many patients for whom blood sampling is difficult, it is easy for many false high values to be generated [64].

These molecular markers of coagulation activation were also subjected to extensive discussions in the JSTH’s committee for creating DIC diagnostic criteria, and in the end it was decided to include markers in the criteria because they had the support of many of the committee members. However, which molecular markers are the best to be included has not yet been settled and will require further discussion. For that reason, it was decided to include all three of these molecular markers of coagulation activation in the new criteria. However, the cutoff values for the molecular markers have yet to be set, and scores were given based on the degree of elevation above the upper limit of the standard range. Using the markers for exclusion diagnosis also seems to make sense. However, if a minus score were assigned when a molecular marker remains within its standard range, there is concern that the diagnosis might be reversed when test results are returned at a later date. Accordingly, this approach was not adopted.

#### Antithrombin (AT) activity

The JSTH’s committee for creating DIC diagnostic criteria discussed many pros and cons regarding incorporation of AT activity into the criteria.

Multiple committee members expressed opinions explaining their being in favor of inclusion of AT activity in the criteria: measurement of AT activity is directly linked to treatment selection (use of AT concentrate for DIC finds AT activity in ≤70 % of cases in Japan); in cases of infection, the sensitivity of DIC diagnosis would be improved by adopting AT activity; the prognosis could be evaluated [46, 65–75].

Negative opinions were also expressed by multiple committee members: it is rare for AT activity to decrease due to DIC mechanisms, and AT activity is not a specific indicator for DIC (the diagnostic specificity would be reduced) [42, 43, 58, 76]; AT activity generally reflects a protein synthesis disorder in the liver or extravasation during inflammation; AT activity correlates with serum albumin [77–80]; the degree of decrease in AT activity differs with the underlying disease; incorporation would complicate the diagnostic criteria.

The committee’s conclusion was as follows: AT activity would be incorporated in the new criteria, but the decision would be re-visited in the future at a time when the results of validation of the new criteria are in hand.

#### Considerations for the fields of obstetrics and pediatrics

In Japan, the obstetric DIC score is frequently used in obstetrics. Obstetric DIC takes a very rapid course and requires prompt diagnosis and treatment of the underlying disease(s) and clinical symptoms. The obstetric DIC score enables early initiation of treatment and is thus extremely useful. It is widely used in Japan (The Japan Society of Obstetrical, Gynecological & Neonatal Hematology; http://www.jsognh.jp/dic/). Moreover, since such DIC-associated markers as FDP, D-dimer, TAT, SF, and F_1+2_ increase even in normal pregnancy [81], DIC cannot be said to be present merely because these markers are elevated. An opinion of committee was expressed that, if the new criteria become diagnostic criteria consisting mainly of blood coagulation and fibrinolysis tests, they will not be able to be applied to obstetric DIC.

Also, an opinion was expressed that, if the new criteria are based on the JMHW criteria, it is highly likely that they will not be able to be applied to diagnosis of DIC in newborn infants. The reason is that some items for coagulation activation and fibrinolytic activation differ greatly between newborns and adults. In addition, since only a limited amount of blood can be drawn from children, especially newborns, it is desirable to keep the number of test items as small as possible. Coagulation activation-related markers such as TAT and SF, are prone to show false high values (leading to misdiagnosis) by ex vivo coagulation for patients for whom blood collection is difficult (such as children) [64]. The consensus reached on the basis of these opinions was that the new criteria would not be applied to newborns.

#### Countermeasures for misdiagnoses

With the JMHW criteria, misdiagnosis readily occurred for patients showing PT prolongation, decreased fibrinogen, and decreased platelet count due to liver failure, as well as patients with elevated FDP and D-dimer when they had liver failure and also massive ascites [36]. It is necessary to make adjustments to avoid misdiagnosis in liver failure cases.

#### Other molecular markers

Once DIC has been diagnosed, other markers are known to be useful for the subsequent steps of disease type classification and pathological evaluation. It was thus decided to also include statements regarding “testing and significance related to DIC diagnosis”.

Various excellent markers are known: PIC and α_2_PI are essential markers for evaluating fibrinolytic activation [7, 9–11]; protein C is an anticoagulant factor for evaluating the prognosis [74, 82–84]; PAI-1 is a fibrinolytic inhibitory factor [73, 85–89]; HMGB-1 is a nuclear molecule [90, 91]; and e-XDP is a fibrin degradation product of granulocyte elastase [92–97].

### The new DIC diagnostic criteria (JSTH’s provisional draft DIC diagnostic criteria)

#### Algorithm of application of the DIC diagnostic criteria (Fig. 1)

**Fig. 1:**
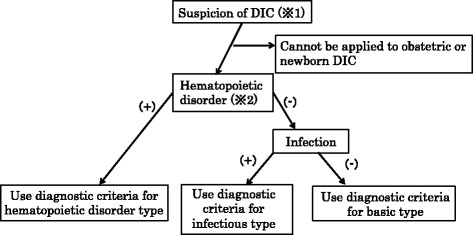
Algorithm for applying the DIC diagnostic criteria. Suspicion of DIC (※1): When there is any underlying disease of DIC (Table 2), an unexplained abnormal laboratory value such as a decreased platelet count, decreased fibrinogen or elevated FDP, or a thrombotic disease such as venous thromboembolism is evident. The new criteria cannot be applied to obstetric or newborn DIC, and for that reason this is shown as the first step in the algorithm. Hematopoietic disorder (※2): A positive (+) judgment is made when it is determined that there is some cause besides DIC for a decreased platelet count, such as bone marrow suppression, bone marrow failure, or platelet destruction or aggregation in the peripheral circulation. For the hematopoietic disorder type, scoring for the platelet count is not performed. Hematopoietic tumors in a state of remission are judged as negative (−). In the absence of a hematopoietic disorder, the possibility of an infection is examined. If an infection is present, the diagnostic criteria for the infectious type are used. Scoring for fibrinogen is not performed for the infectious type. If there is neither a hematopoietic disorder nor an infection, the diagnostic criteria for the basic type are used. When an underlying disease cannot be specified (or there are many), and neither “hematopoietic disorder type” nor “infectious type” applies, the diagnostic criteria for the basic type are used. For example, if an infection accompanies a solid cancer, such that the underlying disease cannot be specified, the diagnostic criteria for the basic type are used

This algorithm should be followed from the time that DIC is suspected.

#### Underlying diseases of DIC

Many underlying diseases of DIC are known. Table 2 shows representative underlying diseases. Obstetric complications and even diseases of the newborn are known to be characteristic underlying diseases of DIC. Specific examples of obstetric complications include placental abruption, amniotic fluid embolism, DIC-type afterbirth bleeding, and eclampsia, while diseases of newborns include neonatal asphyxia, infection, placental abruption, in utero death of one fetus in a multiple pregnancy, respiratory distress syndrome, and intraventricular hemorrhage.

**Table 2:** Underlying diseases of DIC

1. Infections • Sepsis • Other severe infections (of the respiratory organs, urinary tract, biliary system, etc.)	
2. Hematopoietic malignancies • Acute promyelocytic leukemia (APL) • Other acute leukemia • Malignant lymphoma • Other hematopoietic malignancies	
3. Solid cancers (usually advanced cancer with metastasis)	
4. Tissue damage: trauma, burns, heat stroke, rhabdomyolysis	
5. Post-surgery	
6. Vascular-related diseases • Thoracic and abdominal aortic aneurysms • Giant hemangioma • Blood vessel-associated tumors • Collagen disease (cases of vasculitis complications) • Other vascular-related diseases	
7. Liver injury: acute liver failure, acute hepatitis, liver cirrhosis	
8. Acute pancreatitis	
9. Shock	
10. Hemolysis, incompatible blood-type transfusion	
11. Snake bite	
12. Hypothermia	
13. Other	

Note: There are characteristic underlying diseases of DIC in the fields of obstetrics and newborns, but they are not shown in this table because these diagnostic criteria are not applicable to either of those fields

#### Representative underlying diseases and pathologies that must be differentiated

Table 3 shows the representative underlying diseases and pathologies that must be differentiated from DIC.

**Table 3:** Representative underlying diseases and pathologies that must be differentiated

Decreased platelet count	
1.	Enhancement of platelet destruction and aggregation
	• Thrombotic microangiopathy (TMA): thrombotic thrombocytopenic purpura (TTP), hemolytic uremic syndrome (HUS), HELLP syndrome, TMA after hematopoietic stem cell transplantation
	• Heparin-induced thrombocytopenia (HIT)
	• Idiopathic thrombocytopenic purpura (ITP), systemic lupus erythematosus (SLE), antiphospholipid antibody syndrome (APS)
	• Extracorporeal circulation
2.	Pathologies that lead to bone marrow suppression/bone marrow failure
	• Hematopoietic malignancies (acute leukemia, blastic crisis of chronic myelogenous leukemia, myelodysplastic syndrome, multiple myeloma, bone marrow infiltration of malignant lymphoma)
	• Hemophagocytic syndrome
	• Solid cancers (with bone marrow infiltration)
	• Chemotherapy or radiation therapy with bone marrow suppression
	• Bone marrow suppression due to drugs
	• Some viral infections
	• Some blood diseases besides hematopoietic malignancies (aplastic anemia, paroxysmal nocturnal hemoglobinuria, megaloblastic anemia)
3.	Liver failure, cirrhosis, hypersplenism
4.	Sepsis
5.	Bernard-Soulier syndrome, MYH9 disorder (e.g., May-Hegglin disorder), Wiskott-Aldrich syndrome
6.	Dilution
	• Massive bleeding
	• Massive transfusion, massive infusion
	• Pregnancy thrombocytopenia
7.	Pseudo-thrombocytopenia
Elevated FDP	
	1. Thrombosis: deep vein thrombosis, pulmonary thromboembolism
	2. Massive hydrothorax/ascites
	3. Large hematoma
	4. Fibrinolytic therapy
Decreased fibrinogen	
	1. Congenital afibrinogenemia, congenital hypofibrinogenemia, dysfibrinogenemia
	2. Liver failure, malnutrition
	3. Drug-induced: L-asparaginase, corticosteroids, fibrinolytic therapy
	4. False lowering: at the time of administration of drugs with anti-thrombin action (e.g., dabigatran)
Prothrombin time prolongation	
	1. Vitamin K deficiency, oral warfarin
	2. Liver failure, malnutrition
	3. Deficiency or inhibitor of extrinsic coagulation factor
	4. Ingestion of a direct oral anticoagulant
	5. False prolongation: insufficient blood sample volume, addition of an anti-coagulant
Decreased antithrombin activity	
	1. Liver failure, malnutrition
	2. Extravasation due to inflammation (e.g., sepsis)
	3. Degradation by granulocyte elastase (e.g., sepsis)
	4. Congenital antithrombin deficiency
	5. Drug-induced: L-asparaginase
Elevated TAT, SF, or F_1+2_	
	1. Thrombosis: deep vein thrombosis, pulmonary embolism
	2. Some atrial fibrillation

Note: However, DIC may also occur with the above conditions and diseases

#### DIC diagnostic criteria

The first step is to confirm, according to the algorithm (Fig. 1), which diagnostic criteria can be applied to the patient in question, and then proceed to the diagnosis of DIC using Table 4. For the basic type, scoring should be performed using the data for the platelet count, FDP, fibrinogen, PT ratio, AT activity, and coagulation activation-associated molecular markers (elevation of TAT, SF, or F_1+2_). The total score should be calculated, and a diagnosis of DIC should be made for the basic type and the infectious type if the total is 6 points or more and for the hematopoietic disorder type if the total is four points or more. The point for diagnosis of DIC in infectious type is more than 6 points though there is one less item to check with the infectious type as compared to the basic type. This reason is that depression in fibrinogen is hardly observed in DIC caused by infection. However, the point for diagnosis of DIC in infectious type might be modified from 6 points to 5 points after validation of the new DIC diagnostic criteria. Table 4 shows that 3 points are subtracted for liver failure. Scoring for underlying diseases and clinical symptoms was included in the JMHW criteria [22] but omitted from the new criteria.

**Table 4:** JSTH’s provisional draft DIC diagnostic criteria

Classification of type	Basic		Hematopoietic disorder		Infectious	
Platelet count (×10^4^/μl)	>12	0 p			>12	0 p
	8< – ≤12	1 p			8< – ≤12	1 p
	5< – ≤8	2 p			5< – ≤8	2 p
	≤5	3 p			≤5	3 p
	≥30 % decrease w/in 24 h (*1)	+1 p			≥30 % decrease w/in 24 h (*1)	+1 p
FDP (μg/ml)	<10	0 p	<10	0 p	<10	0 p
	10≤ – <20	1 p	10≤ – <20	1 p	10 ≤ – <20	1 p
	20≤ – <40	2 p	20≤ -<40	2 p	20≤ – <40	2 p
	≥40	3 p	≥40	3 p	≥40	3 p
Fibrinogen (mg/dl)	>150	0 p	>150	0 p		
	100< – ≤150	1 p	100< – ≤150	1 p		
	≤100	2 p	≤100	2 p		
Prothrombin time ratio	<1.25	0 p	<1.25	0 p	<1.25	0 p
	1.25≤ – <1.67	1 p	1.25≤ – <1.67	1 p	1.25≤ – <1.67	1 p
	≥1.67	2 p	≥1.67	2 p	≥1.67	2 p
Antithrombin (%)	>70	0 p	>70	0 p	>70	0 p
	≤70	1 p	≤70	1 p	≤70	1 p
TAT, SF or F_1+2_	<2-fold of normal upper limit	0 p	<2-fold of normal upper limit	0 p	<2-fold of normal upper limit	0 p
	≥2-fold of normal upper limit	1 p	≥2-fold of normal upper limit	1 p	≥2-fold of normal upper limit	1 p
Liver failure (*2)	No	0 p	No	0 p	No	0 p
	Yes	˗3 p	Yes	˗3 p	Yes	˗3 p
DIC diagnosis	≥6 p		≥4 p		≥6 p	

p: points

• (*1): For a platelet count of >5 × 10^4^/μL, points will be added if the time-course conditions of decrease are met (no points will be added for a platelet count of ≤5 × 10^4^). The maximum score for the platelet count is 3 points

• For institutions that do not measure FDP (institutions that measure only D-dimer), 1 point will be added if D-dimer increases ≥2-fold the normal upper limit. However, in principle, FDP should also be measured and re-evaluation performed after the results are in hand

• Prothrombin time ratio: If ISI is close to 1.0, INR will also be acceptable (However, there is no evidence supporting recommendation of the use of PT-INR for diagnosis of DIC.)

• Thrombin-antithrombin complex (TAT), soluble fibrin (SF), prothrombin fragment 1+2 (F_1+2_): For blood sampling in difficult cases and route blood sampling, false-high values may increase. Thus, in comparison with elevation of FDP and/or D-dimer, re-testing should be done if TAT and/or SF is markedly elevated. Confirmation is needed even if the results on the same day are not in time

• Regardless of the presence or absence of DIC immediately after surgery, changes in DIC-like markers such as elevation of TAT, SF, FDP, or D-dimer or a decrease in AT, may be observed, and judgment should be made with care

• (*2) Liver failure: Corresponds to “a prothrombin time activity of ≤40 % or an INR value of ≥1.5 due to severe liver dysfunction seen within eight weeks of onset of initial symptoms following liver impairment that develops in a normal liver or a liver that is thought to exhibit normal liver function” (acute liver failure) or “cirrhosis with a Child-Pugh classification of B or C (≥7 points)” (chronic liver failure) that may be viral or autoimmune in origin, drug-induced, or caused by circulatory failure”

Even when DIC is strongly suspected but these diagnostic criteria are not met, there should be no interference with anti-coagulation therapy based on the physician's judgment, but repeated evaluation is necessary

Depending on the value for the platelet count, the score covered a range of 0 to 3 points (the same range as in the JMHW criteria), adding another 1 point if a decrease of ≥30 % is seen within 24 h. However, for a platelet count of ≤5 × 10^4^/μL, no extra point is added even if there is a decrease of ≥30 % within 24 h, so the maximum score for the platelet count is 3 points.

For FDP, fibrinogen, and the PT ratio, the ranges and point scoring methods were the same as those in the JMHW criteria.

The AT activity was not included as a test item in the JMHW criteria, but it has been adopted in the new criteria. A score of 1 point is assigned for an AT activity of ≤70 %.

The coagulation-fibrinolysis system molecular markers were also not used as test items in the JMHW criteria, but they have been adopted in the new criteria. One point is given if these values are ≥2-fold the respective upper limit of the standard range. In the cases of blood sampling being difficult and route blood sampling, values may be increased due to false high values [64], and re-testing should be performed if the TAT and SF data are markedly higher than the degrees of elevation of FDP and D-dimer.

Liver failure includes acute liver failure and chronic liver failure. For acute liver failure, we adopted the terminology used in the diagnostic criteria created by the Ministry of Health, Labour and Welfare’s Intractable Hepato-Biliary Diseases Study Group, which uses the new “acute liver failure” in place of “fulminant hepatitis” [98]. That is, acute liver failure was defined as being caused by viral infection or autoimmune, drug, or circulatory failure, and “liver failure develops in a liver that is normal or is thought to exhibit normal function, and within eight weeks from the initial appearance of symptoms the prothrombin time activity is ≤40 % or the INR value is ≥1.5 due to a high degree of liver dysfunction”. Chronic liver failure was defined as “Child-Pugh classification B or C cirrhosis (≥7 points)” [99].

#### Other tests relating to DIC diagnosis, and their significance

Following diagnosis of DIC, testing should be performed for the markers listed in Table 5, which are useful for disease type classification and pathological assessment.

**Table 5:** Other tests relating to DIC diagnosis, and their significance

Test	Significance
Plasmin-α_2_ plasmin inhibitor complex (PIC)	The higher the values, the greater the fibrinolytic activation
α_2_ plasmin inhibitor (α_2_PI)	This is consumed and decreases due to fibrinolytic activation. However, it is also decreased by liver failure alone, and it is elevated in acute inflammatory diseases.
Protein C (PC)	Low values correlate with a poor prognosis. However, it is also decreased by vitamin K deficiency and/or liver failure alone.
Plasminogen activator inhibitor-1 (PAI-1)	High values in infectious-type DIC correlate with a poor prognosis.
HMGB-1	High values correlate with a poor prognosis.
e-XDP	Both low and markedly elevated values in infectious-type DIC correlate with a poor prognosis.

#### Points of difference between the JMHW criteria and the new criteria

The new criteria make it clear that the algorithm should be used, and the diagnostic criteria should be selected based on the underlying pathology. Even in the JMHW criteria, the scoring method is different for the leukemia and non-leukemia groups, whereas the new criteria make it clear that the diagnostic criteria should be selectively used not only for the hematopoietic disorder type but also the infectious type.

Regarding elimination of the platelet count from the score for the hematopoietic disorder type, the JMHW criteria did the same for the leukemia group, while the new criteria also eliminate fibrinogen from the score for the infectious type.

Although scoring was performed for the clinical symptoms and underlying disease in the JMHW criteria, it was omitted from the new criteria for the above-mentioned reasons.

Points were not added for a temporal decrease in the platelet count in the JMHW criteria, but this was made a 1-point item in the new criteria.

At present, AT activity was tentatively included in the new criteria, and it was decided that validation will be performed at multiple institutions. However, even if AT activity is <70 %, the criteria do not recommend that administration of an AT preparation always be carried out. The overall decision to administer an AT preparation always resides with the attending physician.

Verification must be performed as to which coagulation-fibrinolysis molecular markers are good, but diagnostic criteria that incorporate molecular markers are completely novel.

In the JMHW criteria, as well, 3 points are supposed to be subtracted for liver cirrhosis and chronic hepatitis whose pathology approaches that of liver cirrhosis. However, this has not necessarily been carried out properly in clinical practice, and it had been one of the causes of DIC misdiagnosis. For the new criteria, we took into account that, on that background, the criteria had not conventionally been applied to cases of fulminant hepatitis, and we incorporated a 3-point reduction for liver failure in the table in the new criteria.

## Conclusion

DIC, based on the presence of underlying disease, has a common pathology in that systemic, persistent, marked coagulation activation is caused. However, there are many points of difference in terms of the degree of fibrinolytic activation, the way in which clinical symptoms manifest, and the degree of formation of pathological blood clots. In regard to diagnosis of DIC, it also makes sense to apply diagnostic criteria selectively depending on the pathology.

DIC diagnostic criteria have great significance in regard to patients’ treatment and prognosis. The JMHW criteria have been extensively used in Japan, but it has been pointed out that they have many problems, such as their poor sensitivity in diagnosing DIC due to infections. Meanwhile, the JAAM criteria are effective for diagnosing DIC due to infection, but they are not applicable to all underlying diseases.

In this paper, we have presented the provisional draft DIC diagnostic criteria of the Japanese Society on Thrombosis and Hemostasis. These new criteria have many laudable aspects, including selective use of diagnostic criteria depending on the underlying disease, incorporation of molecular markers and antithrombin, and measures to reduce misdiagnoses. We look forward to further refinement and improvement of these new criteria in the future.

## Abbreviations

DIC: Disseminated intravascular coagulation
ISTH: International Society on Thrombosis and Haemostasis
SSC: Scientific Standardization Committee
PAI-1: Plasminogen activator inhibitor-1
LPS: Lipopolysaccharide
TAT: Thrombin-antithrombin complex
SF: Soluble fibrin
F_1+2_: Prothrombin fragment 1 + 2
PIC: Plasmin-α_2_ plasmin inhibitor complex
FDP: Fibrin/fibrinogen degradation products
α_2_ PI: α_2_ plasmin inhibitor
APL: Acute promyelocytic leukemia
TF: Tissue factor
JMHW: Japanese Ministry of Health and Welfare
JAAM: Japanese Association for Acute Medicine
JSTH: Japanese Society on Thrombosis and Hemostasis
AT: Antithrombin
